# Short-Term Clinical Outcomes of Platelet-Rich Plasma Therapy in Patellofemoral Chondromalacia: Evaluating Pain Reduction and Functional Improvement in a Six-Month Retrospective Study

**DOI:** 10.7759/cureus.99698

**Published:** 2025-12-20

**Authors:** Naif M Alhamam, Turki M Al Dayhur, Hajer E AlSaif, Ziyad S Aldhaif, Nujood A Alhomeni, Bashaer I Almulhim

**Affiliations:** 1 Orthopedics, King Faisal University, Al-Ahsa, SAU; 2 College of Medicine, King Faisal University, Al-Ahsa, SAU; 3 Orthopedic Surgery, King Fahad Hospital, Hofuf, SAU

**Keywords:** anterior knee pain, knee function, non-surgical treatment, patellofemoral chondromalacia, prp therapy

## Abstract

Background: Patellofemoral chondromalacia is a degenerative condition of the knee cartilage, leading to significant pain and functional impairment. Platelet-rich plasma (PRP) therapy has emerged as a promising treatment, leveraging its regenerative and anti-inflammatory properties. This study aimed to evaluate the effectiveness of PRP in reducing pain and improving knee function in patients with patellofemoral chondromalacia at the six-month follow-up.

Methodology: A retrospective cohort study was conducted at Almoosa Specialist Hospital in Al-Ahsa, Eastern Province, Saudi Arabia. Medical records of 103 patients diagnosed with patellofemoral chondromalacia and treated with PRP were reviewed. Patients aged 18 years or older, diagnosed through clinical examination and imaging, and with a six-month follow-up were included. Data were collected on demographics, symptom duration, Outerbridge grade, and pre- and post-treatment scores on the Visual Analog Scale (VAS) and Anterior Knee Pain Scale (AKPS). Descriptive and inferential analyses were performed using IBM SPSS Statistics for Windows, Version 26.0 (IBM Corp., Armonk, New York, United States).

Results: The mean age of participants was 32.86±9.54 years. A total of 80 out of 103 participants (77.7%) were male. PRP treatment significantly reduced VAS scores from 6.42±2.52 to 1.19±1.73 (p<0.001) and improved AKPS scores from 81.17±11.71 to 89.96±7.21 (p<0.001) after six months. While 98.1% of patients showed improvement in VAS scores, 99% demonstrated enhanced AKPS scores. Age significantly influenced outcomes, with older patients showing greater functional improvement. However, the Outerbridge grade remained unchanged across the follow-up period.

Conclusion: PRP injections were associated with a significant reduction in pain and improvement in knee function in patients with patellofemoral chondromalacia, particularly in middle-aged and older populations. Despite significant symptomatic relief, no structural changes in cartilage were observed over six months, as the Outerbridge grade remained unchanged in all patients (mean 2.30±0.79 before and after treatment; p=1.000). These findings support further investigation into the use of PRP as a non-surgical treatment option, warranting additional research to assess its long-term effects and comparative efficacy.

## Introduction

The most common disorder of the knee is patellofemoral chondromalacia, also known as chondromalacia patellae, which means a softening of the cartilage in the undersurface of the patella [1]. Commonly, this shows up with anterior knee pain and crepitus, together with functional limitations, and may ultimately decrease the quality of life in patients [2]. Patellofemoral chondromalacia affects mainly young athletes, especially those involved in sporting activities associated with repetitive knee bending and straightening, such as running, jumping, and cycling. However, in older people, it is part of the degenerative change of aging [3,4].

There are mechanical and biochemical components in the pathophysiology of patellofemoral chondromalacia [5]. The cyclic loading of the patellofemoral joint can cause microscopic chondral trauma, initiating an inflammatory response that ends in chondral degradation. Other predisposing variables considered to start and increase the severity of the condition involve misalignment of the patella, muscle imbalance, and overuse [6].

Classic treatment modalities include physical therapy, nonsteroidal anti-inflammatory drugs, corticosteroid injections, and surgical interventions like arthroscopy or realignment surgical procedures on the patella [7]. In most instances, these measures relieve symptoms without actually addressing the underlying cartilage damage and are thus not very effective in the long term [8].

During the past years, platelet-rich plasma (PRP) therapy has been emerging as one of the most promising regenerative treatments for a variety of musculoskeletal conditions, from tendinopathies and osteoarthritis to cartilage injuries [9]. PRP preparation is an autologous blood product enriched with high platelet and growth factor concentrations; hence, it will enhance tissue healing and modulate inflammation [10]. PRP will induce the proliferation of chondrocytes at the site, increase the production of the extracellular matrix, and enhance angiogenesis, which is generally helpful in the regeneration process of damaged cartilage [11].

While interest in the therapy is high, very few studies exist that exclusively research the efficacy of PRP therapy for patellofemoral chondromalacia. The aim of this study is to bridge this gap in the literature by presenting a clinical outcome evaluation of PRP therapy in patients affected by patellofemoral chondromalacia for a period of six months of follow-up.

## Materials and methods

Study design

This retrospective cohort study was conducted to evaluate the medical records of patients diagnosed with patellofemoral chondromalacia who had undergone PRP injections as a treatment. The study design allowed for the analysis of pre-existing data to identify treatment outcomes and associated factors.

Study setting

The study took place at Almoosa Specialist Hospital, located in Al-Ahsa, Eastern Province, Saudi Arabia, after obtaining approval from the Almoosa Health Group (approval number: ARC-24.9.06). The hospital is a reputable referral center known for its advanced management of musculoskeletal conditions, including the use of PRP therapy.

Study subjects

The study included patients who met the specific inclusion and exclusion criteria. Inclusion criteria comprised patients aged 18 years or older, those diagnosed with patellofemoral chondromalacia confirmed through clinical examination and imaging, and those who had received PRP injections. Only patients with documented follow-up data at least six months post-treatment and who had been treated at Almoosa Specialist Hospital, regardless of the treatment timeline, were included. Exclusion criteria included patients younger than 18 years, those without a confirmed diagnosis of patellofemoral chondromalacia, and those who did not receive PRP injections. Patients with significant coexisting knee pathologies, such as osteoarthritis, ligament injuries, or rheumatoid arthritis, as well as those receiving other concurrent treatments, such as corticosteroid injections, surgery, or physical therapy initiated alongside PRP treatment, were excluded. Additionally, patients with systemic conditions that could affect the healing process, such as diabetes mellitus, autoimmune diseases, or coagulopathy, were not included. Patients with incomplete baseline or follow-up data and those with a history of allergic reactions to PRP components or injection procedures were also excluded.

Sample size and sampling technique

The study assessed the medical records of 130 patients who fulfilled the inclusion and exclusion criteria. These patients had received treatment for patellofemoral chondromalacia at Almoosa Specialist Hospital.

Ethical considerations

Ethical principles were strictly adhered to throughout the study. Patient confidentiality was maintained by anonymizing data and assigning unique codes to each patient record. No identifying information was recorded or disclosed to ensure privacy and compliance with ethical standards.

Data collection plan

Data were collected by trained medical students who reviewed patients' electronic medical records. Relevant information was extracted and initially organized in Microsoft Excel spreadsheets (Microsoft Corporation, Redmond, Washington, United States) before being transferred to the IBM SPSS Statistics software for analysis.

Variables and measurements

The study captured a range of variables to ensure a comprehensive assessment of treatment outcomes. Baseline data included patient demographics such as age and gender, duration of symptoms, Outerbridge grade of chondromalacia, and baseline Anterior Knee Pain Scale (AKPS) and Visual Analog Scale (VAS) scores. PRP treatment details, such as the number of injections administered, were recorded. The specific technical parameters of the preparation and injection protocol were not consistently documented. Follow-up data included AKPS and VAS scores measured at six months post-treatment to evaluate functional improvement and pain reduction.

Data processing and analysis

Data were processed using computer-based methods, including sorting and coding, to ensure accuracy and consistency. Statistical analysis was performed using IBM SPSS Statistics for Windows, Version 26.0 (IBM Corp., Armonk, New York, United States). Descriptive statistics were used to summarize patient demographics, baseline characteristics, PRP treatment details, and follow-up outcomes.

Data sources and study instruments

Electronic medical records served as the primary data source for this study. Validated instruments, including AKPS and VAS, were used to assess functional outcomes and pain levels at baseline and follow-up intervals. These tools ensured reliable and standardized measurements of the treatment's impact.

## Results

Table 1 presents the demographic characteristics of the participants. The mean age of the study population was 32.86 years (SD=9.54), with the largest proportion of participants aged between 19 and 25 years (32%), followed by those aged 36-45 years (28.2%) and 26-35 years (27.2%). Only 12.6% of participants were older than 45 years. The majority of the participants were male (77.7%), with females comprising 22.3% of the study sample. Regarding the affected knee, the right knee was more commonly affected (72.8%) compared to the left knee (27.2%). The duration of symptoms varied widely among participants, with the most common durations being 12 months (29.1%), 24 months (19.4%), and 36 months (17.5%). A smaller percentage of participants reported symptoms lasting six months (17.5%), nine months (4.9%), or seven or eight months (3.9% each).

**Table 1:** Demographic factors of the participants The mean age of participants was 32.86±9.54 years.

Variable	Category	Count	Percentage (%)
Age (years)	19-25	33	32%
	26-35	28	27.2%
	36-45	29	28.2%
	45<	13	12.6%
Gender	Male	80	77.7%
	Female	23	22.3%
Affected knee	Left	28	27.2%
	Right	75	72.8%
Duration of symptoms	4 months	3	2.9%
	6 months	18	17.5%
	7 months	4	3.9%
	8 months	4	3.9%
	9 months	5	4.9%
	12 months	30	29.1%
	24 months	20	19.4%
	36 months	18	17.5%
	48 months	1	1%

Table 2 compares the VAS and AKPS scores of participants before PRP treatment and six months after the intervention. The mean VAS score showed a significant reduction from 6.42 (SD=2.52) before treatment to 1.19 (SD=1.73) at the six-month follow-up, with a mean difference of -5.22 (95% CI: -5.63, -4.81; p<0.001). The AKPS score significantly improved from a mean of 81.17 (SD=11.71) before treatment to 89.96 (SD=7.21) at six months, with a mean difference of 8.79 (95% CI: 7.64, 9.94; p<0.001). There was no change observed in the Outerbridge grade of chondromalacia after treatment, with a consistent mean score of 2.30 (SD=0.79). Paired t-tests showed significant reductions in VAS scores and improvements in AKPS scores after treatment (p<0.001 for both).

**Table 2:** Difference considering VAS and AKPS scores before and six months after PRP Paired t-test was used to compare pre- and post-treatment scores of VAS and AKPS. The Outerbridge grade was analyzed descriptively, as no change was observed. PRP: platelet-rich plasma; VAS: Visual Analog Scale; AKPS: Anterior Knee Pain Scale

Outcome measure	Before PRP treatment		After the six-month follow-up		Mean difference (95% CI)	P-value	t-value
	Mean	SD	Mean	SD			
Outerbridge grade	2.30	0.79	2.30	0.80	No change	-	-
VAS score	6.42	2.52	1.19	1.73	-5.22 (-5.63, -4.81)	<0.001	24.42
AKPS score	81.17	11.71	89.96	7.21	8.79 (7.64, 9.94)	<0.001	-15.08

Table 3 highlights the changes in VAS and AKPS scores after six months of PRP treatment. None of the participants experienced worsening VAS or AKPS scores. Regarding VAS scores, 98.1% of participants reported improvement, while 1.9% showed no change. Similarly, 99% of participants showed improvement in AKPS scores, with only 1% reporting no change. There was no observed improvement in the Outerbridge grade, as 100% of participants retained the same grade post-treatment. These findings suggest that PRP treatment had a significant positive impact on pain and functional outcomes but did not alter the structural grading of chondromalacia within the six-month follow-up period.

**Table 3:** Change in VAS and AKPS scores after six months of PRP PRP: platelet-rich plasma; VAS: Visual Analog Scale; AKPS: Anterior Knee Pain Scale

		Count	Column N %
Change in Outerbridge grade after PRP treatment on the six-month follow-up	Not changed	102	100%
Change in VAS score on the six-month follow-up	Worsen	0	0%
	Not changed	2	1.9%
	Improved	101	98.1%
Change in AKPS score on the six-month follow-up	Worsen	0	0%
	Not changed	1	1%
	Improved	102	99%

Table 4 examines the association between demographic factors and changes in VAS and AKPS scores at the six-month follow-up. Age showed a statistically significant association with changes in both VAS scores (p=0.019) and AKPS scores (p<0.001). Participants aged 36-45 years experienced the largest mean reduction in VAS scores (-6.00; SD=2.04), followed by those aged 26-35 years (-5.54; SD=1.90) and >45 years (-4.85; SD=2.30). Participants aged 19-25 years had the smallest mean reduction in VAS scores (-4.42; SD=2.03). For AKPS scores, participants aged >45 years demonstrated the most significant improvement (mean=13.92; SD=5.45), followed by those aged 36-45 years (mean=10.79; SD=5.58). Participants aged 26-35 years and 19-25 years showed smaller improvements with mean changes of 6.89 (SD=4.79) and 6.64 (SD=5.46), respectively.

**Table 4:** Association between demographic factors and changes in VAS and AKPS scores on the six-month follow-up One-way ANOVA was used to assess associations between age and changes in VAS and AKPS scores. Independent t-tests were used for comparisons across gender and the affected knee. VAS: Visual Analog Scale; AKPS: Anterior Knee Pain Scale

Demographic factor	Group	VAS change (mean±SD)	P-value	Test statistic	AKPS change (mean±SD)	P-value	Test statistic
Age group	19-25	-4.42±2.03	0.019	F=3.43	6.64±5.46	<0.001	F=7.00
	26-35	-5.54±1.90			6.89±4.79		
	36-45	-6.00±2.04			10.79±5.58		
	>45	-4.85±2.30			13.92±5.45		
Gender	Male	-5.12±1.94	0.442	t=0.78	8.28±5.60	0.113	t=-1.63
	Female	-5.57±2.63			10.61±6.53		
Affected knee	Left	-5.68±1.91	0.181	t=1.36	10.04±5.67	0.130	t=-1.54
	Right	-5.05±2.16			8.33±5.91		

One-way ANOVA demonstrated a significant association between age and outcome scores. Independent t-tests were used for gender and knee comparisons. 

## Discussion

This study evaluated the effectiveness of PRP injections in managing patellofemoral chondromalacia and examined the demographic and clinical factors influencing treatment outcomes. The findings indicate significant improvements in pain and functional outcomes, as measured by the VAS and AKPS, respectively, following PRP treatment. However, the Outerbridge grade of chondromalacia remained unchanged during the six-month follow-up period, suggesting that PRP primarily addresses symptomatic relief rather than structural changes in the cartilage.

Pain and functional improvement after PRP treatment

The significant reduction in VAS scores from a mean of 6.42 before treatment to 1.19 at six months (p<0.001) demonstrates the efficacy of PRP injections in alleviating knee pain associated with chondromalacia. Similarly, the improvement in AKPS scores, with a mean increase from 81.17 to 89.96 (p<0.001), indicates enhanced knee function. These results align with previous studies that have reported PRP as an effective therapy for reducing pain and improving function in various knee pathologies, including chondromalacia and osteoarthritis [12-15]. For example, several recent studies highlighted the role of PRP in enhancing symptomatic relief in knee cartilage lesions, emphasizing its regenerative potential through growth factors that promote tissue repair and reduce inflammation [12,14,16-21].

Despite the symptomatic improvements, the lack of change in Outerbridge grade suggests that PRP may not significantly influence cartilage structure within the short-term follow-up period. This finding is consistent with Patel et al., who noted that while PRP can improve clinical outcomes, structural changes in cartilage are less evident within six months of treatment [22]. Longer follow-up periods or advanced imaging studies may be required to assess potential structural benefits of PRP.

Demographic factors and treatment outcomes

The study revealed that age significantly influenced the outcomes of PRP treatment. Participants aged 36-45 years showed the greatest reduction in VAS scores, while those older than 45 years demonstrated the most substantial improvement in AKPS scores. This could be attributed to variations in the healing potential of cartilage and individual response to PRP with age. Younger patients may benefit more from PRP's regenerative effects due to higher biological activity in their cartilage tissue [23-25]. Previous research by Laver et al. supports this observation, indicating that PRP efficacy may vary with age, with middle-aged patients often achieving optimal outcomes [26].

Gender and the affected knee, however, did not show statistically significant associations with changes in VAS or AKPS scores. While male participants had slightly smaller improvements than females, these differences were not clinically significant. This finding contrasts with studies which suggest that hormonal differences might influence PRP efficacy [12,27-30]. The lack of association in this study may reflect a relatively homogenous population or differences in treatment protocols.

Clinical relevance and limitations

The findings of this study underscore the utility of PRP in managing symptomatic patellofemoral chondromalacia. The significant improvements in pain and function suggest that PRP can be a valuable non-surgical intervention, particularly for patients seeking alternatives to invasive procedures. However, the study has several limitations. The retrospective design may introduce selection bias, and the reliance on medical records limits control over data quality. Additionally, the lack of a control group precludes direct comparisons with other treatments, such as corticosteroid injections or physical therapy. Furthermore, the specific technical parameters of the PRP preparation and injection protocol were not consistently documented, which limits the replicability of our findings. This is a significant methodological limitation, as PRP therapy is not standardized, and variations in protocol can substantially influence clinical outcomes, thereby affecting the generalizability of our findings. Future randomized controlled trials are recommended to validate these findings and explore the long-term effects of PRP on cartilage health.

## Conclusions

This study demonstrates that PRP injections are associated with significant improvements in pain and knee function in patients with patellofemoral chondromalacia, with age being an influential factor in treatment outcomes. However, the treatment does not appear to alter cartilage structure within six months. These findings suggest a potential role for PRP as a promising therapy for symptomatic management, though further research is warranted to assess its long-term efficacy and potential for structural cartilage repair.
